# Prevalence of Myopia and Its Associated Factors Among Japanese Preschool Children

**DOI:** 10.3389/fpubh.2022.901480

**Published:** 2022-06-22

**Authors:** Saiko Matsumura, Kazuhiko Dannoue, Momoko Kawakami, Keiko Uemura, Asuka Kameyama, Anna Takei, Yuichi Hori

**Affiliations:** ^1^Department of Ophthalmology, Toho University School of Medicine, Tokyo, Japan; ^2^Dannoue Eye Clinic, Kawasaki, Japan

**Keywords:** spherical equivalent, axial length, axial length to corneal radius of curvature ratio, myopia, parental myopia, screen time

## Abstract

**Purpose:**

To investigate the prevalence of myopia and factors associated with spherical equivalent (SE), axial length (AL), and axial length to corneal radius of curvature (AL/CR) ratio among Japanese preschool children.

**Study Design:**

Prospective observational study.

**Methods:**

This cross-sectional study evaluated subjects aged 4–6 years from a preschool. Non-cycloplegic autorefraction was measured using the Spot Vision Screener, while AL and corneal radius (CR) were measured using the Myopia Master. Parental myopia and environmental factors were investigated using the myopia-related factor questionnaire. The worse eye with higher myopic SE was chosen for analysis, and multiple linear regression models was performed using AL, SE, and AL/CR ratio as dependent variables.

**Results:**

A total of 457 out of 514 participants (239 males, 52.3%) aged 4–6 years (mean 4.77 ± 0.65 years) were included. The mean SE was 0.13 ± 0.63 D, AL was 22.35 ± 0.67 mm, CR was 7.76 ± 0.25 mm, and AL/CR ratio was 2.88 ± 0.72. The overall prevalence of myopia and high myopia were 2.9 and 0.2%, respectively. Multiple regression analysis showed that myopic SE was significantly associated with male sex (β = −0.14, *p* = 0.02) and parental myopia (β = −0.15, *p* = 0.04). Meanwhile, longer AL was significantly associated with older age (β = 0.13, *p* = 0.02), male sex (β = 0.44, *p* < 0.001), parental myopia (β = 0.24, *p* = 0.01), and screen time (including smartphones, tablets, and computers) (>1 h, β = 0.14, *p* = 0.04). A higher AL/CR was significantly associated with older age (β = 0.02, *p* < 0.001), male sex (β = 0.03, *p* < 0.001), ratio and parental myopia (β = 0.03, *p* = 0.02).

**Conclusion:**

The prevalence of myopia and high myopia were 2.9 and 0.2%, respectively, among Japanese preschool children in 2021. Longer AL was associated with older age, male sex, parental myopia, and screen time in children aged 4–6 years. Children with a high risk of myopia can be identified early based on parental myopia information for early prevention.

## Introduction

Myopia has become a critical public health problem worldwide, with a marked increase in its prevalence in developed East Asian countries (1). In Japan, the prevalence of myopia has also increased, from 10% in 6-year-olds and 60% in 12-year-olds in 1999–63% in 6-year-olds and 95% in 12-year-olds in 2017 (2, 3). There have been changes in the rate of myopia not only in school-age children, but also preschool children. Similarly, myopia has increased from 2.3 to 6.3% among preschool children in Hong Kong over 10 years (4). Early onset of myopia has been reported to lead to more myopic refractive error or high myopia later in life (5). Risk factors for the development and progression of school myopia include near work, decreased outdoor time, parental myopia, and education; however, there are few reports of risk factors for preschool myopia. Chua et al. (6) reported that early-onset myopia in 572 preschool children was strongly associated with parental history of myopia [odds ratio (OR) = 4.8; 95% 95 confidence interval (CI): 1.4, 16.6] but not with other environmental factors (near time, outdoor time). An association between increased screen time and myopia has also been recently reported, but this remains controversial (7, 8).

Although a meta-analysis (7) concluded that there is no proven association between digital screen time and myopia, screen exposure in early life could influence preschool myopia. Yang et al. reported that compared to preschoolers without screen exposure, children with younger age at first contact with screens had a significantly higher risk of preschool myopia (9). In addition, the lockdown caused by COVID-19 altered children's life behaviors and increased the progression of myopia. In a report from China, screen time increased 3.14-fold while outdoor time decreased by 1.14-fold in the COVID era compared with the pre-COVID in grade stage 1 (grades 1–6) children, with a correspondingly marked increase in myopia progression over a 6-month period (10).

Amid these major changes in the living environment, it is important to identify children who are at high risk for early onset of myopia and to provide lifestyle guidance and myopia control therapy to slow the progression of myopia. Cycloplegic refraction testing is the gold standard to identify early onset myopia. However, it is difficult to perform due to its longer testing time required and side effects for screening. Given that ocular refractive error is interrelated with both Axial length (AL) and the refractive components of the eye (e.g., cornea and lens), the Axial length to corneal radius of curvature (AL/CR) ratio has been suggested as a proxy for refractive error in the absence of cycloplegic refraction (11). A previous study revealed that the correlation between Spherical equivalent (SE) and AL/CR ratio is stronger than that between AL or corneal radius (CR) alone, which suggests that AL/CR ratio can be a useful marker of the onset of myopia (12). Knowledge of modifiable risk factors associated with myopia may be useful in developing cost-effective strategies to prevent the progression of myopia in Japan. However, studies reporting the prevalence of myopia and its associated risk factors among preschool children in Japan are limited. Thus, this study aimed to investigate the prevalence of early onset myopia as well as the factors associated with longer AL, myopic SE, and longer AL/CR ratio among Japanese preschool children.

## Methods

### Study Design and Subjects

This cross-sectional study evaluated subjects aged 4–6 years from a single preschool located in Kanagawa, Japan. Kanagawa Prefecture is located next to Tokyo and has the second largest population in Japan, 9 million. The kindergarten is located in the urban area of Kawasaki City, the second largest city in Kanagawa Prefecture. Data for all participants were collected from June 10th to June 28th, 2021. Children with chronic eye diseases, such as congenital cataracts, undergoing myopia control therapy, chronic medical disorders, and Down syndrome were excluded. The research protocol was approved by the Ethics Committee of Eiwakai (No. 2021-02) and was conducted in accordance with the tenets of the Declaration of Helsinki. Informed written consent was obtained from all parents.

### Eye Measurements

Refractive status was measured in a non-cycloplegic state for each child using the Spot Vision Screener (SVS) (Welch Allyn, Skaneateles Falls, NY). SVS is a device that measures refraction at 1 m and therefore shows agreement with cycloplegic retinoscope refraction (13, 14). Refractive data from SVS were used in this study because it reduces the effects of accommodations compared to stationary auto-refractometers. Habitual visual acuity was measured using an international standard visual acuity chart in a well-lit room during the day at a 5-m distance by two experienced senior optometrists. Children with prescription glasses had their visual acuity measured on their own glasses. AL and CR were measured using non-contact partial coherence interferometry (Oculus, Myopia Master, Germany).

### Questionnaire on Environmental Factors and Parental Myopia

Parents completed a questionnaire about their children's demographic characteristics; ocular and medical history; and environmental factors, such as time spent on outdoor activities, near work, screen time, and sleeping. Near work activities included homework and pleasure reading. Screens included smartphones, computers, and tablets. Time spent outdoors was defined as the sum of outdoor leisure time and outdoor sports activities time. The average number of outdoor activities per day was calculated using the following formula: (hours spent on weekdays) x 5/7 ± (hours spent on weekends) x 2/7 (15). Parental myopia was defined as the use of glasses or contact lenses for distant viewing by biological parents. All questionnaires were entered twice to ensure their integrity and precision.

### Variable Definition

SE was defined as the spherical power plus half negative cylinder power. Myopia was defined as an SE of −0.50 D or greater, and high myopia was defined as an SE of −6.00 D or greater. Because non-cycloplegic refraction led to overestimation of myopia prevalence, alternative methods have been reported to improve accuracy (16). Thorn et al. suggested that combining non-cycloplegic refraction with visual acuity makes the judgment of myopia more accurate than non-cycloplegic refraction alone (17). Additionally, the criteria for younger children aged 3–6 years should be determined carefully for each age group because the average visual acuity varies with age (16). Hence, following the previous method, myopia was defined as SE ≤ – 0.50 D + uncorrected visual acuity > 0.3 logMAR for children aged 3 years, > 0.2 logMAR for children aged 4–5 years, and > 0 logMAR for children aged 6 years (18). Analysis was performed on the worse eye only in view of the correlation in parental myopia, outdoor time and near work between right and left eyes (2, 18, 19). The worse eye with higher myopic SE was chosen for analysis. If both eyes had the same SE refractive error, the right eye was used in the analysis. The onset of myopia in mothers and fathers was defined by the criteria of the British Birth Cohort Study, with early onset defined as onset at <16 years of age and late onset defined as onset at 16 years of age or older (20).

### Statistical Analysis

One-way analysis of variance was used to compare the mean values of continuous outcomes between the different categories of parental myopic status (none, one, and both). We examined the association of myopic risk factors with SE, AL, and AL/CR ratio using multiple linear regression analysis. Specifically, multiple linear regression models were constructed to evaluate how each myopic risk factor contributes to myopic SE, longer AL, and high AL/CR ratio. All statistical analyses were performed using a commercially available statistical software program (SPSS version 20.0; IBM Corp., Armonk, NY, USA). Statistical significance was set at *P* < 0.05.

## Result

A total of 457 out of 514 participants (participation rate = 88.9%; 239 males, 52.3%) aged 4–6 years (mean 4.77 ± 0.65 years) were included. The mean SE was 0.13 ± 0.63 D; AL, 22.35 ± 0.67 mm; CR, 7.76 ± 0.25 mm; and AL/CR ratio, 2.88 ± 0.72. The clinicodemographic characteristics of the subjects are shown in Table 1. The overall prevalence rates of myopia and high myopia were 2.9 and 0.2%, respectively. The prevalence rates of myopia in subjects aged 4, 5, and 6 years were 1.3% (2/152), 3.8% (9/237), and 3.6% (2/56), respectively. Compared with children without parental myopia, children in whom both parents had myopia had significantly greater myopic SE (*p* < 0.05) and longer AL (*p* < 0.001). Further, AL/CR ratio was higher in children with both myopia parents than in children with one myopia parent (*p* < 0.05) and without parental myopia (*p* < 0.01). Paternal myopia was associated with longer AL (β = 0.23; 95% CI: 0.09, 0.37; *p* = 0.002) and higher AL/CR ratio (β = 0.02; 95% CI: 0.002, 0.03; *p* = 0.02). Maternal myopia was associated with longer AL [β = 0.15; 95% CI: 0.02, 0.29; *p* = 0.04] and higher AL/CR ratio (β = 0.02; 95% CI: 0.006, 0.04*; p* = 0.008) (Table 2). Multiple regression analysis showed that myopic SE was significantly associated with male sex (β = −0.14; 95% CI: −0.25, −0.02; *p* = 0.02) and parental myopia (β = −0.15; 95% CI: −0.31, −0.01; *p* = 0.04) (Table 3). Longer AL was significantly associated with older age (β = 0.13; 95% CI: 0.02, 0.23; *p* = 0.02), male sex (β = 0.44, 95% CI: 0.31, 0.58; *p* < 0.001), parental myopia (β = 0.24; 95% CI: 0.05, 0.42; *p* = 0.01), and screen time >1 h, including smartphones, tablets, and computers (β = 0.14; 95% CI: 0.01, 0.28; *p* = 0.04) (Table 4). AL/CR ratio was significantly associated with age (β = 0.02; 95% CI: 0.01, 0.03; *p* <0.001), male sex (β = 0.03; 95% CI: 0.01, 0.04; *p* < 0.001), and parental myopia (β = 0.03; 95% CI: 0.01, 0.05; *p* = 0.02) (Table 5).

**Table 1:** Demographics and parental characteristics of Japanese preschool children.

	**Baseline**
**Characteristics**	***N* (%), Mean (SD)**
Age (years)	4.77 ± 0.65
4	160 (35.0)
5	240 (52.5)
6	57 (12.5)
Sex	
Male	239 (52.3)
Female	218 (47.7)
Parental myopia	
None	74 (16.3)
One	192 (42.2)
Both	189 (41.5)
Paternal myopia	
No	172 (37.8)
Yes	283 (62.2)
Maternal myopia	
No	172 (37.8)
Yes	283 (62.2)
Outdoor time	
<1 h	57 (13.5)
1–2 h	222 (52.6)
2 h ≤	143 (33.9)
Near work^#^	
<1 h	397 (90.6)
1 h ≤	41 (9.4)
Screen time*	
<1 h	268 (61.4)
1 h ≤	167 (38.6)

N, number; D, diopter; w, week; h, hours. ^#^Including reading and studying. *Including smartphones, computers, and tablets.

**Table 2:** Association of parental myopia with SE, AL and AL/CR ratio among Japanese preschool children.

	**SE (Diopter)**				**AL (mm)**				**AL/CR ratio**			
**Characteristics**	**N**	**Mean (SD)**	β (95% CI)^*^	***P*-value**	**N**	**Mean (SD)**	β (95% CI)^*^	***P*-value**	**N**	**Mean (SD)**	β (95% CI)^*^	***P*-value**
No. of parents with myopia												
0	72	0.26 (1.14)	ref		60	22.14 (0.66)	ref		60	2.86 (0.08)	ref	
1	185	0.12 (0.48)	−0.14 (−0.34, −0.06)	0.12	143	22.32 (0.59)	0.18 (0.00, 0.37)	0.05	143	2.87 (0.07)	0.01 (−0.01, 0.03)	0.37
2	186	0.09 (0.63)	−0.08 (−0.18, 0.01)	0.05	152	22.47 (0.71)	0.17 (0.06, 0.27)	0.002	152	2.90 (0.06)	0.02 (0.01, 0.03)	0.003
Paternal myopia												
No	165	0.17 (0.81)	ref		130	22.21 (0.60)	ref		130	2.87 (0.08)	ref	
Yes	278	0.28 (0.50)	−0.06 (−0.18, 0.06)	0.33	225	22.44 (0.69)	0.23 (0.09, 0.37)	0.002	225	2.89 (0.07)	0.02 (0.002, 0.03)	0.02
Age of onset of paternal myopia (Y)												
≥16	85	0.06 (0.44)	ref		71	22.36 (0.69)	ref		71	2.89 (0.06)	ref	
<16	115	0.17 (0.48)	0.11 (−0.02, 0.24)	0.10	87	22.46 (0.69)	0.10 (−0.12, 0.32)	0.37	87	2.89 (0.07)	0.002 (−0.02, 0.02)	0.87
Maternal myopia												
No	164	0.20 (0.86)	ref		133	22.27 (0.66)	ref		132	2.87 (0.08)	ref	
Yes	279	0.09 (0.44)	−0.10 (−0.22, 0.02)	0.09	222	22.41 (0.67)	0.15 (0.02, 0.29)	0.04	222	2.89 (0.07)	0.02 (0.006, 0.04)	0.008
Age of onset of maternal myopia (Y)												
≥16	68	0.09 (0.50)	ref		56	22.31 (0.71)	ref		56	2.88 (0.08)	ref	
<16	208	0.09 (0.36)	−0.01 (0.11, 0.11)	0.99	163	22.38 (0.67)	0.07 (−0.14, 0.28)	0.52	163	2.89 (0.07)	0.01 (−0.01, 0.03)	0.51

*N, number; Y, years; D, diopter; SE, spherical equivalent; SD, standard deviation; CI, confidence interval; AL, axial length; CR, corneal radius*.

* *Multivariate model includes adjustment for age, sex*.

**Table 3:** Univariate and multivariate analyses of factors associated with SE among Japanese preschool children.

	**SE (Diopter)**				
**Characteristics**	**N**	**Unadjusted-β (95% CI)**	***P-*value**	**Adjusted-β (95% CI)**	***P*-value**
Age (years)	445	0.02 (−0.07, 0.11)	0.62	0.02(−0.07, 0.11)^a^	0.62
Sex					
Male	234	−0.14 (−0.25, −0.02)	0.02	−0.14 (−0.25, – 0.02)^a^	0.02
Female	211	ref		ref	
Parental myopia					
No	72	ref		ref	
Yes	371	−0.16 (−0.31, −0.01)	0.04	−0.15 (−0.31, −0.01)^a^	0.04
Near work					
<1 h	386	ref			
≥1 h	40	−0.01 (−0.21, 0.20)	0.96		
Outdoor time					
<1 h	55	ref			
1 h ≤ Outdoor time <2 h	213	0.08 (−0.10, 0.26)	0.36		
≥ 2 h	142	0.09 (−0.03, 0.21)	0.16		
Screen time^*^					
<1 h	263	ref			
≥1 h	161	0.08 (−0.05, 0.20)	0.24		

*N, number; D, diopter; SE, spherical equivalent; SD, standard deviation; CI, confidence interval; AL, axial length*.

a *Multivariate model includes adjustment for age, sex, and parental myopia*.

* *Screen time including smartphone, computer, and tablet*.

**Table 4:** Univariate and multivariate analyses of factors associated with AL among Japanese preschool children.

	**AL (mm)**				
**Characteristics**	***N***	**Unadjusted β (95% CI)**	***P*-value**	**Adjusted β (95% CI)**	***P*-value**
Age (years)	355	0.13 (0.02, 0.24)	0.02	0.13 (0.02, 0.23)^a^	0.02
Sex					
Male	184	0.44 (0.31, 0.57)	<0.001	0.44 (0.31, 0.58)^a^	<0.001
Female	171	ref		ref	
Parental myopia					
No	60	ref		ref	
Yes	295	0.26 (0.08, 0.44)	0.006	0.24 (0.05, 0.42)^a^	0.01
Near work					
<1 h	305	ref			
≥1 h	36	0.14 (−0.09, 0.37)	0.24		
Outdoor time					
<1 h	40	ref			
1 h ≤ Outdoor time <2 h	170	0.02 (−0.21, 0.24)	0.89		
≥ 2 h	120	−0.03 (−0.27, 0.21)	0.80		
Screen time^*^					
<1 h	206	ref		ref	
≥ 1 h	132	0.18 (0.03, 0.33)	0.02	0.14 (0.01, 0.28)^a^	0.04

*N, number; D, diopter; SE, spherical equivalent; SD, standard deviation; CI, confidence interval; AL, axial length*.

a *Multivariate model includes for age, sex, parental myopia, screen time and outdoor time*.

* *Screen time including smartphone, computer, and tablet*.

**Table 5:** Univariate and multivariate analysis of factors associated with AL/CR ratio among Japanese preschool children.

	**AL/CR ratio**				
**Characteristics**	***N***	**Unadjusted β (95% CI)**	***P*-value**	**Adjusted β (95% CI)**	***P*-value**
Age (years)	334	0.03 (0.01, 0.04)	<0.001	0.02 (0.01, 0.03)^a^	<0.001
Sex					
Male	183	0.03 (0.02, 0.05)	<0.001	0.03 (0.01, 0.04)^a^	<0.001
Female	171	ref		ref	
Parental myopia					
No	59	ref		ref	
Yes	295	0.02 (0.00, 0.04)	0.03	0.03 (0.01, 0.05)^a^	0.02
Near work					
<1 h	304	ref			
≥ 1 h	36	−0.01 (−0.03, 0.02)	0.80		
Outdoor time					
<1 h	39	ref			
1 h ≤ Outdoor time <2 h	170	−0.01 (−0.03, 0.02)	0.47		
≥2 h	120	0.00 (−0.03, 0.02)	0.74		
Screen time^*^					
<1 h	209	Reference			
≥1 h	129	0.01 (−0.01, 0.03)	0.09		

*N, number; D, diopter; SE, spherical equivalent; SD, standard deviation; CI, confidence interval; AL, axial length*.

a *Multivariate model includes adjustment for age, sex, and parental myopia*.

* *Screen time includes for smartphone, computer, and tablet*.

## Discussion

The prevalence of myopia and its associated risk factors among preschool children in Japan are unclear. In this study, myopia and high myopia were prevalent in 2.9 and 0.2% of children aged 4–6 years, respectively. Multivariate analysis revealed that a longer AL was significantly associated with older age, male sex, parental myopia, and screen time. Myopic SE was significantly associated with male sex and parental myopia, and AL/CR ratio was significantly associated with age, male sex, and parental myopia.

Myopia is a disease influenced by environmental factors, and information on the prevalence of myopia is crucial for health policy planning. Our study revealed that, in the absence of cycloplegia, the overall prevalence of myopia was 2.9% among 4–6-year-old Japanese preschool children in urban areas. The prevalence of myopia differs between urban and rural areas, and these differences have been suggested to be possibly related to near-work time, education, outdoor activity level, and economic status. Although there are no reports for the preschool population in Japan, previous reports in China showed myopia was prevalent in 4.1, 1.6, 3.7, and 17.0% of 6-year-old children in Shandong, Guangzhou, Shenzhen, and Hong Kong, respectively (21–23). In comparison of prevalence rates among different studies, the differences in definition of refractive error and urbanity of the area and refractive error measurement techniques should be noted. Zhang et.al reported that the prevalence of myopia (SE ≤ −0.75D) with non-cycloplegic autorefraction was 3.5% in 3–6-year -old children in Hebei Province, China (24). Meanwhile, Li et al. reported that the prevalence of myopia (SE ≤ −1.00 D) with non-cycloplegic autorefraction was 5.9% in 4–6-year-old children in Shanghai (25). However, measurement of refractive error without cycloplegia could overestimate the prevalence of myopia in children.

In the current study, preschool children were examined using a binocular vision system in a non-mydriatic state. The SVS is widely used for refractive examinations in young children and is gradually gaining recognition in both clinical practice and research because of its rapidity, maneuverability, accuracy, and reproducibility (26–28). To reduce the effect of strong accommodation, myopia was additionally assessed by both non-cycloplegic autorefraction and visual acuity in our study. In the report by Wang et al., which assessed myopia in a similar manner, myopia was prevalent in 2.6% and 1.7% of children aged 4 and 5 years, respectively (18). Relatively close values were obtained in the current study. However, the prevalence of myopia at age 6 years was significantly higher at 8.6% in their report than in ours at 3.6%. They also concluded that the prevalence of myopia remained stable before the age of 6 years, but increased with age thereafter. The increase of myopia prevalence after age 6 can be understood by the fact that the bulk of emmetropization occurs in early childhood and is largely complete by age 6 (29). Another reason for this difference can be that their report included data for 6-year-olds in primary school, suggesting that environmental factors in different education systems may have an effect.

Our results revealed that the association of parental myopia, especially in both parents, with myopic SE, longer AL, and higher AL/CR ratio were independent of other environmental risk factors. These results support that children with parental myopia are at high risk of developing myopia. In addition, there was a dose-response relationship between AL and parental myopia. This is consistent with the results of pooled data from children in three population-based studies (19). Parental myopia is associated with a higher ratio of AL/CR ratio and greater myopic refractive error in a cohort study of cycloplegic refraction data from 9,793 children aged 6–72 months (19). Claire et al. revealed that parental myopia represents both a genetic and environmental risk factor. The predictive value for parental myopia (0.67) was as good as that of the genetic risk score (0.67) or environmental risk score (0.69) (30). Normally, genetic testing of young children is not feasible in a clinical setting or at the population level. Meanwhile, it is easy to confirm parental myopia and can be useful in detecting children at risk of myopia before it develops. In the current study, both paternal and maternal myopia were associated with longer AL, and higher AL/CR ratio. Previous studies have shown that parental myopia is associated with various environmental factors. It is possible that parents with higher education tend to provide an environment for children with more reading and studying.

Additionally, an association between myopic SE and other environmental factors, such as outdoor time and near work, was not observed in our study. This may be due to the recall bias of the questionnaires. Overall, as per the present study, genetic susceptibility probably plays a more important role in myopia than do other behavioral factors before school age.

The association between screen time and myopia remains controversial (7–9). A systematic review did not find a significant association between digital screen time and the prevalence of myopia. A recent meta-analysis revealed that smart device screen time alone (OR 1.26) or in combination with computer use (OR 1.77) was significantly associated with myopia in children and young adults (aged 3 months to 33 years). Huang et al. reported the possibility that impact of screen exposure during early childhood on preschool myopia could be diminished by outdoor time for children whose parents have myopia (31). We therefore adjusted for age, gender, parental myopia, and screen time as well as outdoor time in our multivariate analysis. Interestingly, our data showed that screen time was associated with a longer AL after adjusting for age, sex, parental myopia, and outdoor time. The underlying mechanism of the association between screen exposure and myopia has not yet been identified. Some researchers have described screen time as a substitute for near work. Very young children from birth to age 3 may be more sensitive to screen exposure, as very early childhood is an important period for visual development (9). Huang et.al. reported that exposure to fixed screen devices [adjusted odds ratio (AOR) = 2.66] and mobile screen devices (AOR = 2.66) during the early life years (1–3 years) was associated with preschool myopia (31). The World Health Organization recommends <1 h of screen time per day for preschoolers, but our results showed that 38.6% of children were exposed to more than 1 h of screen time. Our results were taken after COVID-19 became a pandemic and may have been affected by the COVID-19 lockdown. From February to the end of May 2020, Japan declared a state of emergency, ordering temporary school closing and voluntary curfews. Subsequently, a quasi-emergency state was declared each time there was a recurring outbreak, and people had to refrain from going out or moving around except when necessary. This has led to behavioral changes in children, such as a decrease in outdoor time and an increase in screen time (32). Our results show that increased screen time is associated with longer AL, which could be augmented by this COVID19-related situations. A report from China showed a 3.14-fold decrease in screen time and a 1.14-fold decrease in outdoor time and faster progression of myopia in elementary school children in the COVID-19 era compared with the pre-COVID-19 era (10). Our results suggest that screen exposure for more than 1 h may pose a risk for myopia in preschoolers.

In the present study, male sex was associated with myopic SE, longer AL, and higher AL/CR ratio among children aged 4–6 years, consistent with previous studies (24, 33). The AL and AL/CR ratio of boys were 0.43 mm and 0.03 higher than that of girls, respectively. These differences may be due to the harmony between eye growth and the body (34). Saw et al. analyzed the height and AL of 1,449 children aged 7–9 years and showed that taller children have a longer AL (35). Ye al. found that in Chinese schoolchildren, personal anthropometric measurements, such as height and weight, maintained an independent relationship with refraction (36). While the difference in height between males and females is considered small compared to school-age children, according to the Japanese Health Statistics Survey, males tend to be taller than females at age 5 (37). Further investigation including physical parameters are needed. Also, age was not associated with SE, but AL and AL/CR ratio in our study. These results may be due to underestimation of SE by non-cycloplegic refraction.

This study has some limitations. First, the possibility of observation and inclusion biases could not be rules out due to the retrospective study design and small sample size. In addition, the data were obtained from only one kindergarten in Kanagawa Prefecture, which is an urbanized and densely populated city. This might limit the generalizability of the findings to other populations in Japan. Second, refraction was determined by non-cycloplegic autorefraction, which may result in misclassification of refractive error with an overestimation of myopia. However, myopia was assessed by combining refractive correction and visual acuity to reduce this effect. Third, the data on environmental and genetic factors were provided by parents and collected with a self-administered questionnaire, which might introduce recall bias. However, the findings of this study are based on previous use of these questionnaires and are consistent with the findings of previous studies.

## Conclusion

The prevalence of myopia and high myopia were 2.9 and 0.2%, respectively, among Japanese preschool children in 2021. Longer AL was associated with older age, male sex, parental myopia, and screen time in Japanese children aged 4–6 years. Parental myopia, especially in both parents, is associated with a greater risk of myopic SE, longer AL, and higher AL/CR ratio in preschool-aged children. Our study underlines the importance of obtaining an accurate family history of myopia to identify at-risk children before they develop myopia and to raise awareness on lifestyle-based myopia prevention from an early stage. These risk factors should be considered when developing screening and intervention guidelines for preschool children.

## Data Availability Statement

The raw data supporting the conclusions of this article will be made available by the corresponding author on reasonable request without undue reservation.

## Ethics Statement

The studies involving human participants were reviewed and approved by the Ethics Committee of Eiwakai. Written informed consent to participate in this study was provided by the participants' legal guardian/next of kin.

## Author Contributions

SM and KD: study concept and design. SM, KD, KU, AK, and AT: data collection. SM and MK: analysis and interpretation of data and statistical analysis. SM and YH: drafting of the manuscript. YH: critical revision of the manuscript for important intellectual content. All authors have reviewed the manuscript.

## Funding

This research was supported by JSPS KAKENHI Grant Number JP19K09961 to YH and Toho University School of Medicine Project Research Funding Grant Number 21-01 to SM.

## Conflict of Interest

The authors declare that the research was conducted in the absence of any commercial or financial relationships that could be construed as a potential conflict of interest. The handling editor CCL declared a past co-authorship with the author SM.

## Publisher's Note

All claims expressed in this article are solely those of the authors and do not necessarily represent those of their affiliated organizations, or those of the publisher, the editors and the reviewers. Any product that may be evaluated in this article, or claim that may be made by its manufacturer, is not guaranteed or endorsed by the publisher.

## Abbreviations

AL: axial length
CR: corneal radius of curvature
AL/CR: axial length to corneal radius of curvature
OR: odds ratio
SE: spherical equivalent.
